# Effects of a Chinese Herbal Medicine, Guan-Jen-Huang (*Aeginetia indica* Linn.), on Renal Cancer Cell Growth and Metastasis

**DOI:** 10.1155/2012/935860

**Published:** 2011-10-18

**Authors:** Yu-Huei Liu, Meng-Luen Li, Meng-Yu Hsu, Ya-Yueh Pang, I-Ling Chen, Ching-Kuei Chen, Sai-Wen Tang, Hsuan-Yuan Lin, Jung-Yaw Lin

**Affiliations:** ^1^Institute of Biochemistry and Molecular Biology, College of Medicine, National Taiwan University, Taipei 100, Taiwan; ^2^Department of Medical Genetics and Medical Research, China Medical University Hospital, Taichung 404, Taiwan; ^3^Graduate Institute of Integrated Medicine of Chinese Medicine, China Medical University, Taichung 404, Taiwan

## Abstract

*Aeginetia indica* Linn. (Guan-Jen-Huang, GJH), a traditional Chinese herb, has the potential to be an immunomodulatory agent. The purpose of this study was to explore the effect of GJH in the treatment of renal cancer. Concentration-effect curves for the influence of GJH on cellular proliferation showed a biphasic shape. Besides, GJH had a synergistic effect on cytotoxicity when combined with 5-fluorouracil (5-FU)which may be due to the alternation of the chemotherapeutic agent resistance-related genes and due to the synergistic effects on apoptosis. In addition, treatment with GJH extract markedly reduced 786-O cell adherence to human umbilical vein endothelial cells (HUVECs) and decreased 786-O cell migration and invasion. In a xenograft animal model, GJH extract had an inhibitory effect on tumor cell-induced metastasis. Moreover, western blot analysis showed that the expression of intercellular adhesion molecule-1 (ICAM-1) in 786-O cells was significantly decreased by treatment with GJH extract through inactivation of nuclear factor-*κ*B (NF–*κ*B). These results suggest that GJH extract has a synergistic effect on apoptosis induced by chemotherapeutic agents and an inhibitory effect on cell adhesion, migration, and invasion, providing evidence for the use of water-based extracts of GJH as novel alternative therapeutic agents in the treatment of human renal cancer.

## 1. Introduction

Renal cell carcinoma (RCC) is the most common solid lesion found within the kidney and accounts for approximately 90% of renal malignancies [1]. Despite improvements in diagnostic techniques and treatment strategies, currently there is no evidence that the use of targeted therapies, alone or in combination with adjuvant treatments, treats localized RCC and improves overall survival. However, 4 large randomized adjuvant clinical trials are ongoing and will address the feasibility and efficacy of treatment in localized RCC [2, 3]. In addition, currently licensed target agents are cost-ineffective and have a lot of common side effects. Therefore, a more potent alternative agent is required to achieve acceptable clinical and oncological outcomes.

Metastasis, a common feature of RCC, is a multistep process that leads to the development of secondary tumors. Preventing the occurrence of any of these steps would prevent metastasis [4]. During the metastatic cascade, primary tumor cells digest their surrounding extracellular matrix, migrate through interstitial spaces, and enter the blood or lymphatic vessels where they are carried to distant organs [5]. The adhesion of circulating tumor cells to the microvascular endothelium of organs at distant sites is an important step in metastasis. Once lodged in the target organs, these cells migrate into the interstitial spaces and continue to grow and develop a secondary tumor, or they metastasize [6]. Thus, the adhesion, migration, and invasion of cancer cells provide many potential targets for therapeutic intervention.

Increasing evidence suggests that some medicinal herbs may decrease the risk of malignancies. Studies have shown that phytochemicals contained within herbs are promising chemopreventive agents. *Aeginetia indica* Linn. (Chinese name “Guan-Jen-Huang”, GJH), a root parasitic plant, has been used as folk medicine in Taiwan and other countries to treat chronic liver diseases, cough, and arthritis. The seed extract of GJH induces potent antitumor immunity [7–9]. This formula is used as health food even today, and several studies have demonstrated the antitumor effect of a 55-kDa protein isolated from the seed of GJH [8, 10–12]. However, the effects of GJH herbal extract in human cancers remain to be determined.

In the present study, we report that a water-based extract of GJH inhibits tumor growth and metastasis. The treatment of 786-O renal carcinoma cells with GJH resulted in a synergistic effect on 5-fluorouracil (5-FU)-induced apoptosis, inhibition of cell migration and invasion, and reduction in cell adherence to endothelial cells. The molecular mechanisms involved in these effects include the downregulation of chemotherapeutic agent resistance-related genes and intercellular adhesion molecule-1 (ICAM-1) expression, in parallel with the reduction of nuclear factor-*κ*B (NF-*κ*B) expression and activation.

## 2. Materials and Methods

### 2.1. Plant Extracts

GJH plants were approved and extracted by Sun Ten Pharmaceutical Company (Taipei, Taiwan). In brief, dry plant materials were finely ground, and the extracts were prepared by boiling 250 g of plant material in 1250 mL of water for 30 min. The extracts were concentrated to 250 mL with an evaporator at room temperature. The extract was then centrifuged at 3000 rpm for 10 min and filtered through a 0.45-mm syringe filter. Stock solutions at a concentration of 1 g/mL were stored at −20°C until use.

### 2.2. Cell Lines

The RCC cell line 786-O was obtained from the American Type Culture Collection and maintained in RPMI 1640 (Invitrogen) supplemented with 10% fetal bovine serum (FBS, Invitrogen), 2 mM l-glutamine (Invitrogen), and 100 *μ*g/mL penicillin-streptomycin (Invitrogen). Human umbilical vein endothelial cells (HUVECs) were obtained from the Bioresource Collection and Research Center and maintained in medium 199 (Invitrogen) supplemented with 20% FBS, 25 U/mL heparin (Sigma), and 30 *μ*g/mL ECGS (Sigma). All cells were maintained in a humidified atmosphere of 5% CO_2_ at 37°C.

### 2.3. Cell Proliferation Assay

786-O cells were seeded in 96-well plates in serum-reduced medium (1% FBS) containing various concentrations of GJH (0–100 mg/mL) at a density of 2 × 10^4^ cells/mL per well for 24 h. To investigate the combined effect of GJH and anticancer drugs, 786-O cells were seeded in 96-well plates at 5 × 10^3^ cells/mL per well and incubated for 24 h. On the following day, 100 *μ*L aliquots containing GJH and/or the anticancer drugs 5-FU, cisplatin, or paclitaxel (all from Sigma) were added to each well and the cells were cultured for a further 48 h. The number of viable cells was estimated by measuring the conversion of the tetrazolium salt MTT to formazan crystals. After incubation with MTT for 4 h, formazan crystals were solubilized with DMSO and quantified spectrophotometrically by measuring the absorbance at 590 nm with a reference wavelength of 650 nm.

### 2.4. Combination Index Analysis

The combined effects of GJH and anticancer drugs were quantified using a combination index (CI) method developed by Chou and Talalay [13]. This method involves plotting dose-effect curves for each agent and their combination, using the median-effect equation: *fa/fu = (D/Dm)m,* where *D* is the dose of the drug, *Dm* is the dose required for a 50% cytotoxic effect (equivalent to CC_50_), *fa* and *fu* are the affected and unaffected fractions (*fa* = 1 − *fu*), respectively, and *m* is the exponent signifying the sigmoidicity of the dose-effect curve. The relative concentrations of GJH and the anticancer drugs, determined as (concentration)/(CC_50_ value), were used for analysis. The values of *Dm* and *m* were calculated first. The CI used for analysis of the drug combinations was determined by the equation for mutually nonexclusive drugs that have different modes of action: CI = (*D*)1/(*D*x)1 + (*D*)2/(*Dx*)2 + (*D*)1(*D*)2/(*Dx*)1(*Dx*)2, where (*D*)1 and (*D*)2 are relative concentrations of drugs 1 and 2, and *x* is the percentage of inhibition. Combination indices CI < 1, CI = 1 and CI > 1 indicate synergistic, additive, and antagonistic effects, respectively.

### 2.5. Annexin V-Binding Assay

786-O cells were seeded at a density of 1.0 × 10^6^ cells/well and incubated with the indicated doses of GJH, 5-FU, GJH + 5-FU, or 3% H_2_O_2_ (as a positive control) for 48 h. Cells were then collected by centrifugation (1000 ×g for 5 min), washed twice with Annexin V-binding buffer (PBS containing 2.5 mM CaCl_2_), and resuspended in the same buffer. After that, 200 *μ*L of the cell suspension was incubated with 5 *μ*L of biotin-conjugated Annexin V (biotin-Annexin V; Biovision) for 5 min at room temperature in the dark. The cells were then washed twice with Annexin V-binding buffer and fixed in 200 *μ*L of 2% formaldehyde in PBS for 15 min. After washing twice with PBS, the fixed cells were incubated with horseradish peroxidase (HRP)-conjugated streptavidin (eBioscience) for 30 min at room temperature. The biotin-Annexin V bound to phosphoserine (PSer) exposed on the cell surface was detected spectrophotometrically using the HRP substrate 3,3′,5,5′-tetramethylbenzidine (TMB; eBioscience), by measuring the absorbance at 450 nm with a reference wavelength of 650 nm.

### 2.6. Real-Time Quantitative RT-PCR

Total RNA was extracted using TRIzol reagent (Invitrogen) in accordance with the manufacturer's instructions and further digested with DNase I (Promega). The integrity of the RNA was confirmed before quantitative (q) RT-PCR analysis. Then, 1 *μ*g of RNA was reverse transcribed in the presence of SuperScript II RT (Invitrogen) and oligo(dT) primers (Invitrogen) at 42°C for 1 h followed by incubation at 70°C for 15 min to inactivate the enzymes. Amplification of the cDNA was performed using SYBR Green PCR Master Mix (ABgene) and analyzed with the iCycler iQ Real-Time PCR Detection System (Bio-Rad). Primers were designed by the Beacon Designer 4 program (Premier Biosoft International) and sequences are listed in Table 1. The PCR conditions comprised an initial denaturation at 95°C for 15 min, followed by 40 cycles at 95°C for 10 s and 60°C for 45 s. A dissociation procedure was performed to generate a melting curve for confirmation of the amplification specificity. The results were normalized to glyceraldehyde 3-phosphate dehydrogenase. The relative levels of gene expression are represented as −ΔCt = (Ct_gene_ − Ct_reference_), and the fold change in gene expression was calculated by the 2(−ΔΔCt) method (where Ct is cycle threshold) as described previously [14].

### 2.7. Antibodies and Western Blotting

Antibodies against intact or cleaved forms of of poly (ADP-ribose) polymerase (PARP), *β*-catenin, cyclin D1, and ICAM-1 were obtained from Cell Signaling Technology. Antibodies against NF-*κ*B p65 and p50 subunits and Sp-1 were purchased from Santa Cruz Biotechnology. A primary antibody against total actin and goat anti-mouse and goat anti-rabbit HRP secondary antibodies were from Chemicon. Total cell lysates extracted with lysis buffer (50 mM Tris-HCl [pH 8.0], 150 mM NaCl, 0.5% sodium deoxycholate, 0.1% sodium dodecyl sulfate [SDS], and 1% NP-40) containing a protease inhibitor cocktail, or nuclear fraction extracted using the NE-PER Nuclear Protein Extraction Kit (Thermo Scientific), were analyzed. The protein concentration was determined using a BCA protein assay kit (Thermo Scientific). Proteins were separated by 10% SDS-polyacrylamide gel electrophoresis and transferred to a polyvinylidene fluoride membrane (Immobilon-P, 0.45 mm; Millipore, Billerica, MA, USA) using NA-1512 Semi-Dry Transfer apparatus (NIHON EIDO). The membranes were blocked with 5% skim milk in Tris-buffered saline containing 1% Tween 20 (TBST, pH 7.4) at room temperature for 30 min and incubated overnight at 4°C with primary antibody. The membranes were washed 4 times with TBST for 10 min each at room temperature and incubated with HRP-conjugated secondary antibody for 1 h at room temperature. The membranes were then washed 4 times with TBST. Proteins were visualized using an enhanced chemiluminescence (ECL) kit and western blotting detection reagents (GE Healthcare Life Sciences) and exposed to X-ray film (Fuji, Tokyo, Japan). Each band was quantitatively determined using the Image J program (http://rsb.info.nih.gov/). The densitometry readings of the bands were normalized to actin expression.

### 2.8. Cell Motility Assay

786-O cells were left untreated or treated with 2.5 or 5.0 mg/mL of GJH for 24 h at 37°C, seeded in a 6-well plate, and grown overnight to confluency in serum-containing medium. The confluent cell layer was scratched with a p200 Eppendorf pipette tip. After 24 h, the closure of the scratch wound was photographed at 400x magnification under a phase contrast microscope (Olympus).

### 2.9. Cell Invasion Assay

786-O cells (1 × 10^5^ cells) were resuspended in 300 *μ*L medium containing 1% FBS with various concentrations of GJH and were then seeded into Transwell inserts (8 *μ*m pore; Millipore) precoated with growth factor-reduced Matrigel (1 *μ*g/mL; BD Biosciences). Complete medium was added to the lower chamber. After incubation for 24 h, invasive cells were fixed, stained, and quantified in 3 random fields (100x magnification) per insert.

### 2.10. Cell-Cell Adhesion Assay

HUVECs were seeded onto 6-well plates and left to grow for 48 h before experiments. 786-O cells were treated with GJH (1.3–5.0 mg/mL) in serum-reduced medium (1% FBS) for 6 h and then labeled with the fluorescent dye BCECF/AM (Sigma) at 37°C for 30 min. The fluorescence-labeled 786-O cells were pelleted and resuspended (3 × 10^4^ cells) in medium 199 with 10 mM N-2-hydroxyethylpiperazine-N′-2-ethanesulfonic acid buffer (M199H) and added onto the HUVECs layer. After 30 min, cell suspensions were discarded and the adhered labeled 786-O cells were gently washed with M199H. The number of labeled cells was measured using SigmaGel 1.0 (Jandel Scientific). Analyses were repeated 3 times over the same region, and the results presented are the mean values of 3 independent experiments.

### 2.11. Animal Experiments

All animal experiments were conducted according to the regulations approved by the Institutional Animal Care and Use Committee of the College of Medicine, National Taiwan University. Female nonobese diabetic/severe combined immunodeficient mice (6–8 weeks old) were obtained from the Animal Center of National Taiwan University. 786-O cells (1 × 10^6^ cells) were suspended in 200 *μ*L of growth factor-reduced medium and inoculated intravenously into the tail vein of mice weighing 20 to 25 g (*n* = 10). Two days after injection, the mice were orally administered either water or GJH (25 g/kg) daily and weighed every other day (*n* = 5 for each group). After oral administration of GJH for 30 days, the mice were sacrificed and their lungs were excised and weighed to estimate tumor content.

### 2.12. Electrophoretic Mobility Shift Assay (EMSA)

786-O cells in serum-reduced medium (1% FBS) were treated with GJH (1.3–5.0 mg/mL) for 6 h. Nuclear extracts were prepared using the NE-PER Nuclear Protein Extraction Kit (Thermo Scientific). Detection of NF-*κ*B was performed with a biotin-labeled oligo probe containing the proximal NF-*κ*B recognition site, which spans the region of the human ICAM-1 promoter: 5′-GGGAGCCCGGGGAGGATTCCT-3′ [15]. For competition experiments, excess cold oligonucleotide probe (2-fold excess and 4-fold excess without biotin label, presented as 2 × URE and 4 × URE, resp.) was added 15 min before addition of the labeled probe. Supershift assays were performed with 1 *μ*g of the antibody against p65 incubated for 30 min at 4°C after addition of the probe. The reaction products were analyzed via 5% nondenaturing polyacrylamide gel electrophoresis using 12.5 mM Tris, 12.5 mM boric acid, and 0.25 mM EDTA (pH 8.3), for 4-5 h at 280–300 V/10–12 mA. The gels were transferred and blocked as described above. After incubating at room temperature for 1 h with HRP-conjugated streptavidin (eBioscience), the membranes were washed and visualized as described above.

### 2.13. Luciferase Reporter Assay

A total of 1 × 10^5^ 786-O cells were plated in a 6-well plate for 24 h. Following transfection with 0.1 *μ*g of a NF-*κ*B-responsive luciferase reporter for 6 h, the cultures were replated into a 96-well plate at a density of 2 × 10^4^ cells/mL per well. The cells were grown in standard medium for another 18 h followed by treatment with GJH alone or in combination with TNF-*α* for 6 h. Luciferase activity was measured using a Luciferase Assay Kit (Stratagene) according to the manufacturer's instructions, using a luminometer (Berthold LB960) and an integration period of 60 s.

### 2.14. Statistical Analysis

The Image J program (http://rsb.info.nih.gov/) was used for quantization of the expression fold in western blot or EMSA analyses. The fold increase of the indicated proteins was determined by normalizing to actin or Sp1 when they could be detected. SPSS 12.0 for Windows (SPSS Inc.) was used to analyze all data. A two-tailed paired-samples Student's *t*-test was used for statistical analysis of comparative data from 2 groups. A *P* value <0.05 was considered statistically significant.

## 3. Results

### 3.1. Effect of GJH on the Growth of 786-O Renal Carcinoma Cells

**Figure 1:**
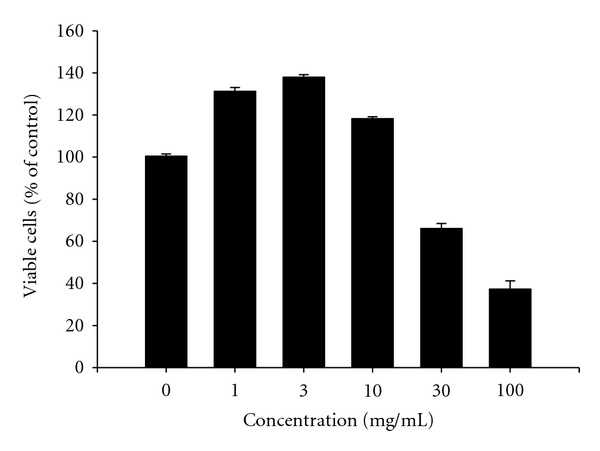
Biphasic effects of GJH on cell viability in 786-O cells. Cells were incubated with GJH (1–100 mg/mL) for 24 h. The viability of cells was determined by MTT assay. Low concentrations (1–10 mg/mL) of GJH increase whereas high concentrations (>10 mg/mL) repress cell proliferation. The mean (SD) is shown from at least 3 separate experiments. ***P < 0.001.

To evaluate the effect of GJH on renal cancer cells *in vitro*, 786-O renal carcinoma cells were exposed to 1–100 mg/mL of GJH for 24 h. Cell viability was then determined by MTT assay. After 24 h of treatment, low doses (1.0, 3.0, and 10.0 mg/mL) of GJH increased 786-O cell proliferation, whereas high doses (>10 mg/mL) caused a decrease in 786-O proliferation (Figure 1). Among the concentrations tested, 3.0 mg/mL GJH was the most effective in stimulating 786-O cell proliferation. The growth of 786-O cells decreased in a dose-dependent manner. The value of the 50% cytotoxic concentration (CC_50_) for 24 h of treatment was determined to be 35 ± 0.5 mg/mL. These findings indicate that low concentrations of GJH induce the proliferation of 786-O cells, while high doses of GJH are cytotoxic for 786-O cells.

### 3.2. Effects of GJH Supplementation on the Anticancer Action of 5-FU In Vitro

**Figure 2:**
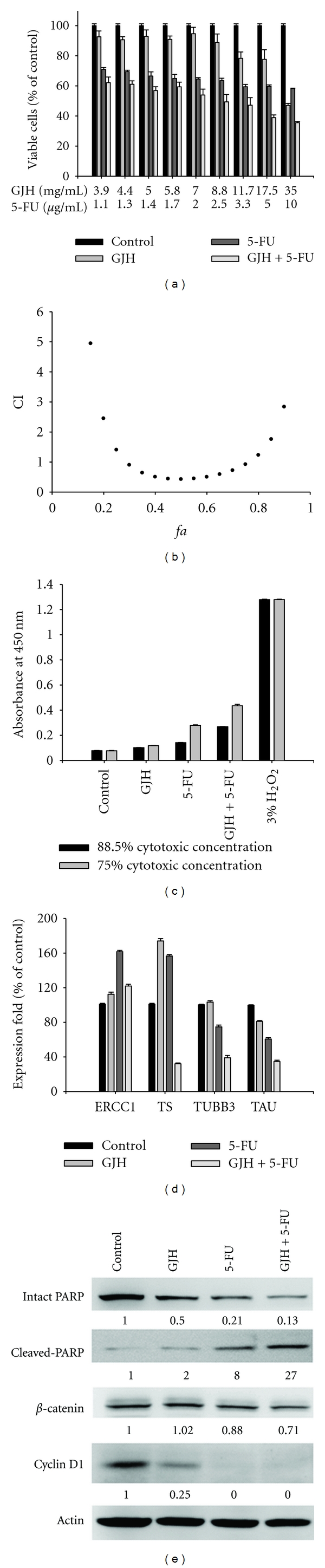
Synergistic effect between GJH and 5-FU on 786-O cancer cell apoptosis. (a) A dose-response survival curve for GJH and the chemotherapeutic agent 5-FU in 786-O cells. (b) CI values at different levels of growth inhibition effect (*fa*). (c) GJH (CC_12.5_ and CC_25_) enhances the apoptotic response to 5-FU (CC_12.5_ and CC_25_, resp.) as demonstrated by Annexin V-binding assay. (d) Combination treatment with GJH and 5-FU upregulates the expression of ERCC1 but downregulates the expression of TUBB3, Tau, and TS1 as shown by real-time quantitative RT-PCR. (e) Western blot analysis indicates that GJH (CC_25_) enhances the 5-FU (CC_25_)-induced cleavage of PARP, as well as 5-FU-induced reduction in expression of *β*-catenin and cyclin D1. The mean (SD) is shown from at least 3 separate experiments.

To explore the potentially useful combination of GJH with chemotherapeutic agents commonly used in renal cancer therapy, we assessed the interaction between GJH and several chemotherapeutic agents in 786-O cells. The synergistic analysis indicated that GJH had a synergistic effect on the cytotoxicity of 5-FU in a relatively broad dose inhibition range (30–75% fraction affected in 786-O cells; Figures 2(a) and 2(b)), while no synergistic interaction between GJH and cisplatin or paclitaxel was observed (data not shown). Biotin-Annexin V binding was detected in target cells as an index for the translocation of PSer from the inner to the outer monolayer of the cell surface membrane (flip-flop) that happens during the early stages of apoptosis. The 3% H_2_O_2_ treated group showed a relatively high intensity of binding (absorbance, ~1.3 at 450 nm) that served as a positive control. As shown in Figure 2(c), the negative control (PBS treatment) showed ~3.3% of the apoptotic response seen in the 3% H_2_O_2_ group. Although GJH (at 12.5% and 25% cytotoxic concentration, CC_12.5_ and CC_25_, resp.) slightly enhanced apoptotic responses (5.2% and 6.5% of the H_2_O_2_ group, resp.), the combination of GJH and 5-FU (CC_12.5_ or CC_25_ for both treatments) induced significantly stronger apoptotic responses (18.7% and 32.1% of the H_2_O_2_ group, resp.) when compared to 5-FU alone (CC_12.5_ and CC_25_ were 8.55% and 19.4%, resp. of the H_2_O_2_ group). 

We also investigated the influence of GJH on chemotherapeutic agent-associated gene expression. GJH in combination with 5-FU could significantly suppress the expression levels of chemotherapeutic agent resistance-related genes in 786-O cells. Real-time quantitative RT-PCR showed that GJH significantly enhances the alternation of chemotherapeutic agent resistance-related genes: upregulation of excision repair cross-complementing gene 1 (ERCC1) and downregulation of thymidylate synthase 1 (TS), class III *β*-tubulin (TUBB3), and microtubule-associated protein tau (Tau), respectively, (CC_25_ of GJH + CC_25_ of 5-FU was more effective than either CC_25_ of GJH or CC_25_ of 5-FU alone; Figure 2(d)). In addition, western blot analysis indicated that GJH enhanced 5-FU-induced cleavage of PARP, as well as 5-FU-induced reduction in expression of *β*-catenin and cyclin D1 (CC_25_ of GJH + CC_25 _of 5-FU was more effective than either CC_25_ of GJH or CC_25_ of 5-FU alone; Figure 2(e)). These results indicate that a useful synergistic interaction exists between GJH and 5-FU in 786-O renal cancer cells. Possible mechanisms for this synergy may include the alternation of chemotherapeutic agent resistance-related genes and synergistic effects on apoptosis.

### 3.3. Antimetastatic Effect of GJH in 786-O Cells *In Vitro* and *In Vivo*

**Figure 3:**
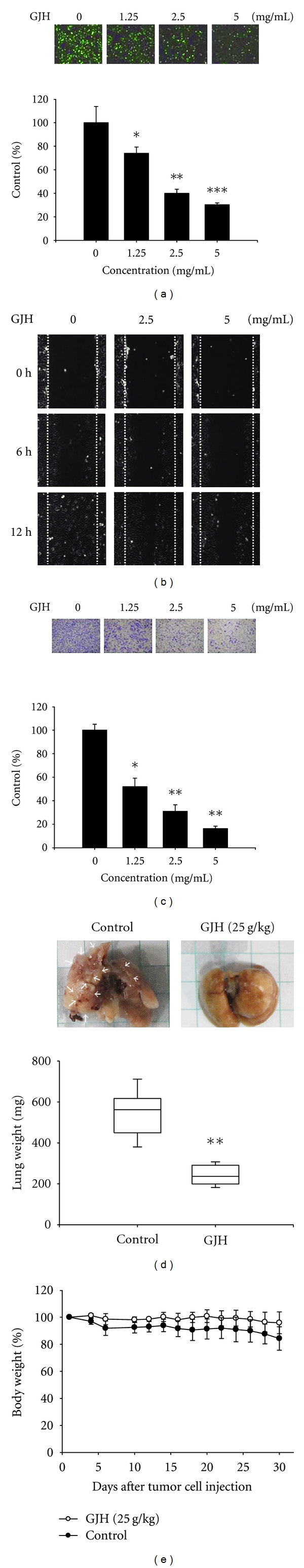
Antimetastatic effect of GJH on 786-O cells. GJH (1.3–5.0 mg/mL) significantly reduced the (a) adhesion of 786-O cells to HUVECs, the (b) migration and (c) invasion of 786-O cells. (d) Animal experiments showed that oral administration of GJH (25 g/kg per day for a total of 30 days) significantly reduces metastatic tumor nodules in the lungs as demonstrated by lung weight. (e) There was no significant difference in body weight between mice treated with (open circle) and without (closed circle) GJH. The mean (SD) is shown from at least 3 experiments. *P < 0.05,**P < 0.01,***P < 0.001.

Cell adhesion and migration are important factors to consider when investigating the metastatic potential of cancer cells. Adhesion to extracellular matrices is considered to be a pivotal step in the invasive process of metastatic cells. To investigate the potential effect of GJH on metastasis, we first examined the adhesion of 786-O cells to confluent monolayers of HUVECs in 6-well plates after exposure of the cells to GJH for 6 h. A significant reduction in adhesion was observed in GJH (1.3–5.0 mg/mL)-treated cells when compared with the control cells (69.7% reduction after 5.0 mg/mL GJH treatment; Figure 3(a); *P* < 0.001). The inhibition of 786-O cell migration was examined using a wound healing assay, and the results are shown in Figure 3(b). Phase-contrast images were taken at 6 and 12 h. Longer (12 h) incubation with GJH (2.5 and 5.0 mg/mL) led to greater inhibition of cell migration in 786-O cells. A Matrigel invasion assay was performed to determine the anti-invasive effect of GJH in 786-O cells. After 24 h of incubation, GJH (1.3–5.0 mg/mL)-treated cells showed a decreased level of invasion compared to control cells (83.7% reduction after 5.0 mg/mL GJH treatment; Figure 3(c); *P* < 0.01). 

The effects of GJH on cell metastasis were then examined. Animal experiments showed that oral administration of GJH (25 g/kg per day) significantly reduced metastatic tumor nodules in the lungs during a 30-day follow-up period (542.0 ± 124.5 mg versus 243.0 ± 52.6 mg in control group and GJH treated group, resp.; Figure 3(d); *P* < 0.01). In addition, the dosage used *in vivo* did not have any significant toxic effects and did not significantly alter body weight during the 30-day experimentation period (79.6 ± 8.1% versus 94.9 ± 11.3% in control group and GJH treated group, resp.; Figure 3(e); *P* > 0.05). Taken together, these results support the idea that GJH inhibits renal cancer metastasis *in vitro* and *in vivo*.

### 3.4. Downregulation of ICAM-1 Expression and Reduction in NF-*κ*B Transcriptional Activity Are Involved in GJH-Mediated Anticancer Effects

**Figure 4:**
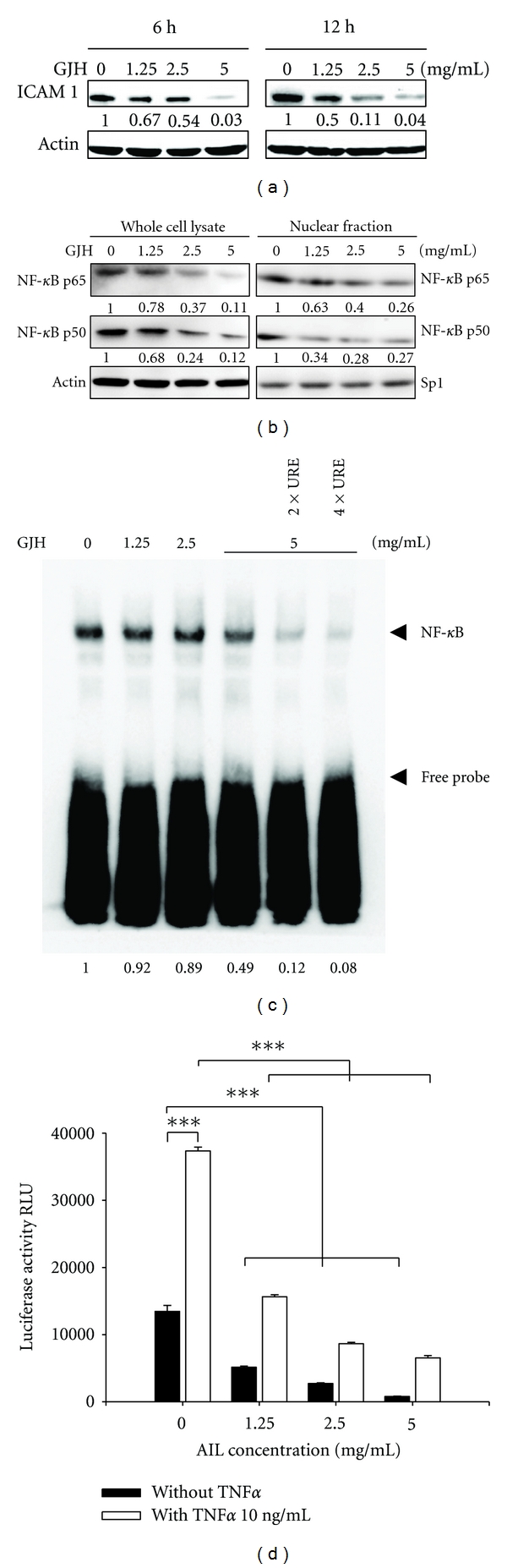
GJH downregulates the expression of ICAM-1 through reduction of the expression and transcriptional activity of NF-*κ*B. (a) GJH significantly reduces the expression of ICAM-1 in both dose- and time-dependent manners, as shown by western blot analysis. (b) Western blotting reveals that GJH reduces the amounts and the nuclear translocation of NF-*κ*B p65 and p50 subunits in the cells. (c) GJH reduces the DNA-binding activity of NF-*κ*B to ICAM-1 as shown by EMSA analysis. (d) GJH downregulates TNF-*α*-dependent and TNF-*α*-independent ICAM-1 expression in part through NF-*κ*B as demonstrated by the luciferase reporter assay. The mean (SD) is shown from at least 3 separate experiments. ***P < 0.001.

It is known that cancer cell-endothelial cell interactions are regulated in part by the expression of specific adhesion molecules on the cell surface, such as ICAM-1. Therefore, the expression of ICAM-1 was examined in GJH-treated and untreated cells. Western blot analysis indicated that GJH significantly reduces the expression of ICAM-1 in both dose- and time-dependent manners (Figure 4(a)). Since aberrant regulation of the transcription factor NF-*κ*B and its signaling pathways are involved in cancer development and progression [16, 17], and the induction of ICAM-1 involves activation of NF-*κ*B, the effects of GJH on NF-*κ*B were examined. Whole cell lysates or nuclear extracts of 786-O cells with or without GJH treatment were prepared, and the expression and the nuclear translocation of NF-*κ*B protein were analyzed. Western blot analysis showed that the amounts of p65 and p50 NF-*κ*B subunits in the nuclear fraction were significantly reduced in both whole cell lysate and nuclear fraction of 786-O cells after GJH treatment (Figure 4(b)). Meanwhile, by investigating the NF-*κ*B binding activity to the *ICAM-1* promoter, EMSA analysis showed that the DNA-binding activity of NF-*κ*B to the *ICAM-1* promoter was decreased by GJH treatment (50.1% reduction after 5.0 mg/mL GJH treatment; Figure 4(c)). To quantify binding activity, a luciferase reporter containing the NF-*κ*B binding region was introduced into 786-O cells. NF-*κ*B-induced luciferase activity was reduced by 61.8%, 79.8%, and 94.1% after treatment with 1.3, 2.5, and 5.0 mg/mL GJH, respectively, (Figure 4(d), black bar). TNF-*α* (10 ng/mL) increased the activity of NF-*κ*B by 2.77-fold; this was reduced by 58.1%, 76.8%, and 82.5% by 1.3, 2.5, and 5.0 mg/mL GJH, respectively, (Figure 4(d), white bar). It is known that TNF-*α* plays a role in the induction of ICAM-1expression via activation of NF-*κ*B. Therefore, these results imply that GJH downregulates TNF-*α*-dependent and TNF-*α*-independent ICAM-1 expression, in part by reducing the expression and inactivating the transcriptional activity of NF-*κ*B.

## 4. Discussion

Although many anticancer drugs are used clinically, they generally induce strong cellular cytotoxicity and related side effects. A cancer drug with little or no toxicity to normal cells is required. Many studies have shown that GJH has a variety of therapeutic effects [7–12]. However, the effects of water-based extracts of GJH in human cancers have not been determined. In the present study, we explored the anticancer effects of a water-based extract of GJH. The molecular mechanisms of GJH action involve the downregulation of chemotherapeutic agent resistance-related genes and ICAM-1 expression, in parallel with the reduction of NF-*κ*B expression and activation in 786-O renal carcinoma cells. This results in a synergistic effect on 5-FU-induced apoptosis, inhibition of cancer cell migration and invasion, and adherence of cancer cells to HUVECs. To the best of our knowledge, the present study is the first to demonstrate the therapeutic potential of GJH in human renal carcinoma. According to the compatibility of Chinese herbal medicine, GJH belongs to a monarch formula, which may need an assistant or a guide formulas to form a prescription. The potential clinical applications of GJH may include enhancement of drug targeting as well as decreased side effects in renal cancer patients.

The adhesion molecule ICAM-1 plays an important role in the regulation of cellular inflammatory responses [8] and transduces several intracellular signal transduction pathways. Its expression is activated by immunotherapeutic agents such as TNF-*α*, alone or in combination with other interleukins, in patients with renal cancer [18–25]. The present study shows that GJH can reduce cancer cell motility* in vitro *and *in vivo*. In addition, the inhibition of oncogenic functions including the adhesion of cancer cells to endothelial cells, cell migration, and cell invasion by GJH occurred concurrently with the reduction of ICAM-1 expression. This indicates that ICAM-1 expression may be of functional importance in cancer cell adhesion and motility. Our preliminary data indicated that a high content of apigenin was detected in the water-based extract of GJH (Figure  1 of Supplementary Material available at doi: 10.1155/2012/935860), and in GJH-induced cytoskeleton rearrangement through downregulation of Rac 1 expression (Figure  2 of Supplementary Material). These results suggest that apigenin may be one of the major active anticancer components of GJH. Although the detailed contents of GJH and their molecular mechanisms need to be investigated further, these results provide information related to the ability of GJH extracts to arrest cell motility in renal cancer cells, supporting its potential to inhibit metastatic renal tumors. 

On the other hand, the death and growth of cells are balanced by the opposing processes of cellular apoptosis and proliferation, respectively, under normal conditions. Inhibition of uncontrolled cell proliferation and/or enhancement of cellular apoptosis may help to maintain normal cellular homeostasis and decrease the chances of neoplastic progression. Our results demonstrate a biphasic effect of GJH on the growth of cultured human renal cancer cells and only showed significant inhibition of growth at higher concentrations. Some phytochemicals, such as curcumin and apigenin, also show biphasic effects in cultured cells; low concentrations stimulate cell proliferation, whereas high concentrations are cytotoxic [26, 27]. One reason for this may be the involvement of distinct kinases or growth factors at different concentrations of GJH. Although the reason for this differential effect of GJH on renal cancer cells remains to be determined, the current study evaluated the synergistic effect of GJH in combination with 5-FU, but not cisplatin and paclitaxel, in apoptosis. Similarly to the way that the importance of food synergy (the perspective of evaluating whole foods rather than single food components) has been clarified [28, 29], the synergistic effect supports the use of GJH in combination with 5-FU during cancer therapy. 

In conclusion, the present study shows that GJH has a synergistic effect on 5-FU-mediated apoptosis and inhibits cell adhesion and motility. The results of this study support the potential of GJH as a useful chemotherapeutic agent for the treatment of renal cancer. Its clinical application warrants further investigations into the molecular mechanisms and beneficial effects of GJH.

## Supplementary Material

Supplementary figure 1: The apigenin in GJH extracts was identified and quantified using a method based on reversed-phase high performance liquid chromatography coupled to mass spectrometry.
Supplementary figure 2: GJH-induced cytoskeleton rearrangement involves downregulation but not inactivation of Rac1 as shown by confocal microscope analysis, Rac activation assay and Western blot analysis.

## Figures and Tables

**Table 1:** Oligonucleotide sequences used in real-time qRT-PCR.

Gene	Oligonucleotide sequence
ERCC1	5′-GGGAATTTGGCGACGTAATTC-3′
	5′-GCGGAGGCTGAGGAACAG-3′
TUBB3	5′-GCGAGATGTACGAAGACGAC-3′
	5′-TTTAGACACTGCTGGCTTCG-3′
Tau	5′-TGACACGGACGCTGGCCTGAA-3′
	5′-CACTTGGAGGTCACCTTGCTC-3′
TS1	5′-GGCCTCGGTGTGCCTTT-3′
	5′-GATGTGCGCAATCATGTACGT-3′
GAPDH	5′-TCAACGACCACTTTGTCAAGCT-3′
	5′-GTGAGGGTCTCTCTCTTCCTCTTGT-3′
