# Supplementary material for: Effects of a Chinese Herbal Medicine, Guan-Jen-Huang (Aeginetia indica Linn.), on Renal Cancer Cell Growth and Metastasis
Source: Evid Based Complement Alternat Med. 2011 Oct 18;2012:935860. doi: 10.1155/2012/935860 (PMC3199064; doi:10.1155/2012/935860)

Supplementary Data

Materials and methods

HPLC Analysis

The analytical HPLC system employed consisted of a high-performance liquid chromatograph coupled with a UV-vis multiwavelength detector (Thermo Scientific). The analytical data were evaluated using a data processing system (ChromQuest 5.0 Chromatography Data System). The separation was achieved on a Waters Spherisorb 5 μm ODS2 4.6 × 250 mm column at ambient temperature. The mobile phase consisted of water with 1% glacial acetic acid (solvent A) and water with 6% glacial acetic acid (solvent B). The gradient used was 100% A, 0-10 min; 100% B, 10-16 min and post-time, 100% A, 10 min before next injection. The flow rate was 0.5 mL/min, and the injection volume was 20 μL. The monitoring wavelength was 280 nm. The identification of apigenin compound was based on a combination of retention time, spectral and mass spectrum matching. The marker apigenin was present at 6.8 mg/g in AIL ± 10%.

Confocal microscopy

786-O cells in serum-reduced medium (1% FBS) were seeded into 6-well plates with 22 × 22 mm coverslips and treated with various concentrations of AIL at 37˚C for 6 h. The cells were washed with PBS three times, fixed with 4% paraformaldehyde at room temperature for 10 min and then permeabilized with 1% saponin for 10 min and blocked with 5% skim milk for 30 min. Alexa568-phalloidin (Invitrogen) was used to stain the F-actin and Hoechst 33258 (Invitrogen) was used to obtain the nucleus for 15 min at room temperature. Images were analyzed using a Leica TCS SP5 Spectral Confocal System.

Rac activation assay

pGEX-2T containing PAK1 (p21-activated kinae 1)-PBD (p21 binding domain) was provided by Dr. Zee-Fen Chang. Glutathione agarose beads coated with GST-PAK1-PBD were prepared as described previously (1, 2). Cells lysed in a buffer containing 50 mM Tris-HCl, pH 7.5, 1% Triton X-100, 0.5% sodium deoxycholate, 0.1% SDS, 500 mM NaCl, 10 mM $MgCl_2$, and protease inhibitor were divided into two part. One part is used directly for Western blot as input control and another is used to immunoprecipitation (IP). For IP, cell lysates were incubated with GST-PAK1-PBD beads for 1 hour at 4˚C. After washed with the lysis buffer mntioned above, the immunoprecipitated proteins were resolved by SDS-PAGE, transferred to PVDF membrane, and detected by Western blotting with Rac1 antibody.

References

1. Benard V, Bohl BP, Bokoch GM. Characterization of rac and cdc42 activation in chemoattractant-stimulated human neutrophils using a novel assay for active GTPases. *J Biol Chem* 1999;274:13198-13204. Available at http://www.ncbi.nlm.nih.gov/entrez/query.fcgi?cmd=Retrieve&db=PubMed&dopt=Citation&list_uids=10224076.

2. Benard V, Bokoch GM. Assay of Cdc42, Rac, and Rho GTPase activation by affinity methods. *Methods Enzymol* 2002;345:349-359. Available at http://www.ncbi.nlm.nih.gov/entrez/query.fcgi?cmd=Retrieve&db=PubMed&dopt=Citation&list_uids=11665618.

Supplementary Figures

Supplementary figure 1. Identification of apigenin in GJH extracts. (a) HPLC-UV chromatogram of GJH. (b) HPLC-UV chromatogram of apigenin. (c) Electrospray (ESI) product ion mass spectrum of apigenin (MW = 270); precursor ion: m/z 271 representing the protonated apigenin; collision energy: 35 eV; collision gas: argon at 270 mPa; the most abundant protonated molecular ion m/z 271.

Supplementary figure 2. GJH-induced cytoskeleton rearrangement involves downregulation of Rac1. (A) GJH promotes the cytoskeleton rearrangement in 786O cells. Nucleus (left) was stained by Hoechst 33258, while F-actin (meddle) by Alexa568-phalloidin. Merged images are shown in the right panels. Bars, 20 μm. (B) GJH reduces Rac1 expression but not activation significantly. The total lysates (Input) and pull-downed lysates (immunuprecipitate) were subjected to Western blot analysis for Rac1.

Supplementary figure 1.

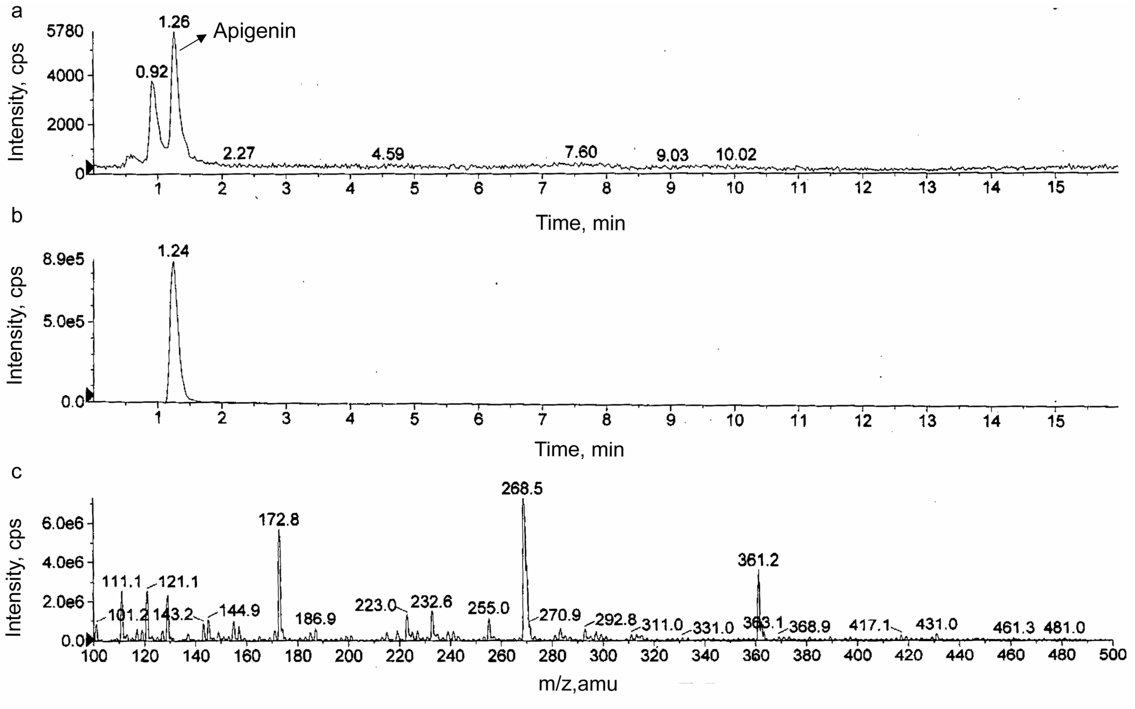

Supplementary figure 2.

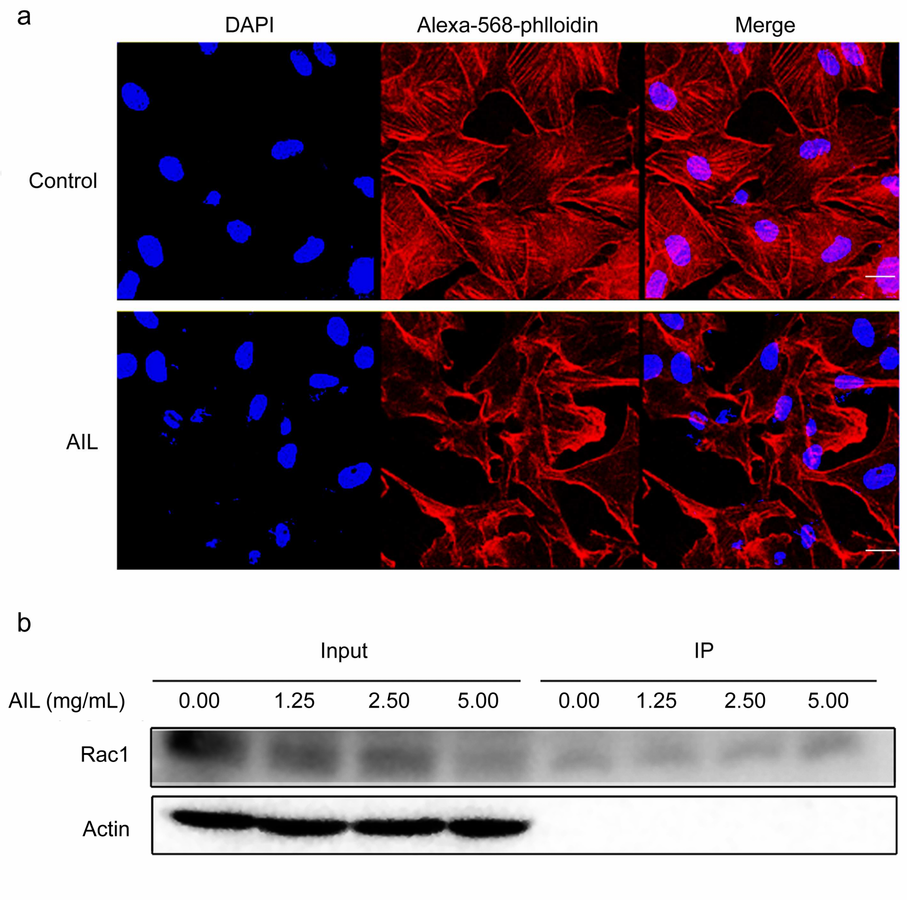

Supplement: Supplementary file 1 — Supplementary figure 1: The apigenin in GJH extracts was identified and quantified using a method based on reversed-phase high performance liquid chromatography coupled to mass spectrometry. Supplementary figure 2: GJH-induced cytoskeleton rearrangement involves downregulation but not inactivation of Rac1 as shown by confocal microscope analysis, Rac activation assay and Western blot analysis. [file 935860.f1.pdf]
